# Hygiene of Young Girls

**Published:** 1904-11

**Authors:** 


### HYGIENE OF YOUNG GIRLS.*

By the expression "young girls" is meant those approaching puberty. At this period too little attention is given in the country to both mental and physical growth; many are compelled to do hard work in factory or farm with poor food. They may become muscular, strong and well developed in their bony structures, but they do not have handsome and graceful figures nor the intellectual training so much desired. On the other hand, in some localities—New England for example—the mental training is often far beyond the brain capacity. Each is objectionable and in great measure preventable. What modern civilization demands is an even development of body and mind. What can be done to aid in securing it?

We must begin with the parents—for heredity plays a very important part, especially in the matter of the nervous system. Insanity, alcoholism, epilepsy, and the like, I need hardly dwell on them, as they are so well known. When, however, we have to deal with that condition we call neurasthenia and hysteria, it is very difficult. Naturally, as a rule, it is the mother who transmits this, and by her lack of mental balance strengthens and intensifies this condition in her daughter, and if the father is also weak and "soft," and permits and encourages this state of affairs, it is a very difficult thing indeed for us to manage such cases. The constant suggestion of the mother has its effect on the daughter, and she is finally imbued with the thought that she is "nervous and can not help it." All our efforts to improve the condition are negatived by the mother with the statement, "Oh, she can not do that, I never could either when I was her age. Her nerves are

*Abstract of paper read by J. H. Carstens, M.D., Detroit, Mich., Professor of Gynecology in the Detroit College of Medicine, at the American Medical Association.

too weak, my nerves were too weak also," and so on. We might just as well give up unless we can get the young girl away from home and put her under different surroundings. She must be entirely taken away from the influence of the mother and have the care of a trained nurse, who will teach her and who, by constant suggestions in another direction, can show that she is not nervous, not so weak and delicate as her mother thinks. And in the course of a few years a wonderful change can take place.

Again, there is the mother who constantly suggests, month after month, that there must be something the matter with her daughter, with her menstrual function, until it is impressed on the young girl's mind that there really is some trouble, and she will always think in the future that her pelvic organs are weak. So I could go on with numerous other points.

If we now turn and go to the opposite extreme, among the poor and poverty-stricken, we find that children are made to work very hard, to carry burdens, to lift their little brothers and sisters, perhaps have improper food, and thus often produce injuries of all kinds: rupture, curvatures, chlorosis, etc. Ignorance on the part of the mother is very frequently the trouble. In all these cases it is the mother that must be treated. We must get her to understand that we must reform her, and show her how to properly treat, manage and train her daughter so she will grow up strong and healthy. And in nearly all cases with the right kind of argument and teaching we can get the mother's mind so stirred up that we will have comparatively little trouble in managing these cases properly. Nevertheless, this class of young girls must often work at quite an early age, and we should regulate the kind of work that each one is capable of doing. Fortunately a great deal is being done by the State in the enactment of laws regulating the lines of work. This kind of laws should be encouraged, as they tend to improve the health of the young girls. First, in regulating the hours of labor. Second, proper ventilation. Third, in proper toilet facilities. Fourth, in the opportunity of getting food at proper intervals.

This brings us to the question of hygiene. In all these cases, rich or poor, we must regulate diet above all other things. Medication also sometimes will be required, but comparatively little. The various gymnastic sports should be encouraged, but regulated by the physician. This is the only way we are able to reach some people and make them take the necessary exercise. The one trouble with most of these is that they are lopsided, will develop only certain parts of certain muscles and parts of the body, while it is so essential to have a systematic and symmetrical development of the body. We are not always able to reach our ideals with stubborn and selfish patients, but we must do the best we can and always strive for it.

Trying, therefore, to guide the young girl to strong and vigorous motherhood, it is not necessary to pay any attention to the menstrual function. That will come without trouble unless there are organic defects and changes which will have to be looked after and remedied, but that does not concern us here.

If we now consider the mental development we have the greatest difficulty. If their physical weaknesses are marked, the mental training should cease or be checked. In this we will have comparatively little trouble. The rich will be willing to do so because they seem nearly always anxious to stop their studies or postpone them on the slightest provocation. This holds good especially with the so-called "shoddy." With the poor and the middle classes we shall have our greatest trouble. If the young girl has been animated by ambition to be something in the future, to be able to support herself, and perhaps often to assist her parents and younger brothers or sisters, we will have a difficult task if such a person is physically weak. If we allow her to go on with her mental work her physical condition will probably become still worse. She is liable to have an undeveloped uterus and conditions of the system produced by it; poorly developed lungs, weak heart and arteries, flabby muscles, feeble digestion, malnutrition of the nervous system and final collapse. Physically collapsed sometimes she will become when the goal is reached for which she struggled: the diploma, the teacher's certificate or whatever it may be. She has a mind educated for requirements of that position, and a body feeble and weak, and if she marries she will be a burden to the poor husband, in fact ultimately will probably be divorced or a subject to a series of operations, which will be done to remedy her defects.

One of the greatest difficulties is that these people learn with difficulty. They have not the right kind of brains; they have to study hard until late at night to get their lessons. This intensifies and hastens the physical downfall. Such persons should stop mental work for a year or two and do some other kind of work. When the body is more developed they can resume their mental efforts. They often object to this, but we can manage them all right if we insist on it. People with the right kind of brains and quick perception can continue studies and with a proper amount of physical training they will get along all right.

The question of age in all these cases cuts no figure in my opinion. Either mentally or physically some girls are further advanced at twelve than others at eighteen.

In conclusion, let me say that hygiene of young girls is one of the most difficult problems for the gynecologist to solve. There are no mathematical problems, no rule that is even elastic within a certain limit, but every case almost is absolutely distinct and requires to be studied by itself. The hereditary predisposition, the surroundings, the future course, the position in life, the means at their disposition, the development of the different parts of the body, or the lack of development of certain parts, the assimilation and elimination of the various organs, the brain and the kind of brain, how it should be developed in one direction or another, and a thousand and one things must be considered in each individual case. Added to this there must be that fine tact and that absolute control over individuals, so that we can not only manage the young patient, but also those around her, be it the mother, father, sister, cousin or aunt. We must get them all under our control ere we can expect to accomplish anything.

We are able to teach the students but very little. We can give them a few hints, but the cases are so diverse and complicated that every medical man must work out each case for himself.

